# Effect of chronic lung diseases on mortality of prevariant COVID-19 pneumonia patients

**DOI:** 10.3389/fmed.2022.957598

**Published:** 2022-10-13

**Authors:** Hatice Kilic, Emine Arguder, Aysegul Karalezli, Ebru Unsal, Rahmet Guner, Bircan Kayaslan, İmran Hasanoglu, İhsan Ates, Musa Civak, Esmehan Akpınar, Ebru Parlak, Filiz Sadi, Yasin Kocaman, Sibel Günay, Esra Metan, Mukremin Er, Aynil Dalkıran, Habibe Hezer, Hülya Ergüden, Zeynep Hancıoğlu, Ayşe Kalem, Fatma Eser, Adalet Aypak, Esragül Akıncı, Selma Karahmetoğlu, Emin Gemcioglu, Emra Kalkan, Osman İnan, Abdulrezzak Yilmaz, Bagdagul Güler, Esra Çopuroğlu, İşil Turan, Derya Gökmen, Serhat Hayme, Aziz Ahmet Surel

**Affiliations:** ^1^Department of Pulmonary Medicine, Faculty of Medicine, Ankara Yildirim Beyazit University, Ankara, Turkey; ^2^Department of Infectious Disease, Faculty of Medicine, Ankara Yildirim Beyazit University, Ankara, Turkey; ^3^Department of Internal Medicine Disease, Faculty of Medicine, Ankara University of Health Science, Ankara City Hospital, Ankara, Turkey; ^4^Department of Internal Medicine Diseases, Ankara City Hospital, Ankara, Turkey; ^5^Department of Chest Diseases, Ankara City Hospital, Ankara, Turkey; ^6^Department of Infectious Disease, Faculty of Medicine, University of Health Science, Ankara, Turkey; ^7^Department of Anesthesia, Ankara City Hospital, Ankara, Turkey; ^8^Department of Biostatistics, School of Medicine, Ankara University, Ankara, Turkey; ^9^Department of General Surgery, Ankara City Hospital, Ankara, Turkey

**Keywords:** COVID-19, lung, chronic, mortality, COPD, lung cancer, interstitial lung disease

## Abstract

**Background:**

The aim of the study is to assess the effect of chronic lung disease on mortality in patients hospitalized with the diagnosis of prevariant COVID-19 Pneumonia compared to patients without chronic lung disease.

**Research design and methods:**

A cohort of 1,549 patients admitted to the pandemic clinic with a COVID-19 Pneumonia diagnosis was analyzed. Group 1 and Group 2 were compared in terms of the treatment they received, admission to intensive care, mortality and follow-up parameters.

**Results:**

The patient group with COVID-19 and lung disease consisted of 231 participants (14.91%) (Group 1). The patient group with COVID-19 but without lung disease had 1,318 participants (85.19%). Group 1 cases were found to receive more oxygen therapy and mechanical ventilation than Group 2 cases (*p* ≤ 0.001), Following univariate and multiple logistic regression analyses, it was determined that patients with chronic lung disease had a 25.76% higher mortality risk [OR: 25.763, 95% CI (Lower-Upper) (2.445–271.465), *p* = 0.007].

**Conclusion:**

It was found that chronic lung disease contributed significantly to mortality in this study. Among chronic lung diseases, Chronic Obstructive Pulmonary Disease (COPD), lung cancer and interstitial lung diseases (ILDs) were shown to be more effective than other chronic lung diseases in patients with prevariant COVİD-19 population.

## Introduction

COVID-19 has had tremendous negative effects worldwide. According to a WHO report dated 2 May 2020, 3.267.184 cases had been diagnosed with COVID-19 and 229.971 deaths (7.03% fatality rate) had occurred (1, 2). The United States (US) became the epidemic center of this pandemic, reporting an estimated 956.000 cases of COVID-19 infections, and the largest concentration was in New York City and its surrounding areas (approximately 35% of all the US infections) (3, 4). According to the Health Ministry Data of Turkey, however, the total number of cases was 124.375 in Turkey, and there were 3.336 deaths (case fatality rate 2.68%) as of 2 May, 2020 (1).

With such alarming consequences in its short history, the infection has the common symptoms of respiratory symptoms, fever, cough, and dyspnea. Also, pneumonia, severe acute respiratory infection, kidney failure, and even death may develop in more serious cases.

According to the WHO’s COVID-19 report of the People’s Republic of China, death cases were generally individuals with advanced age or concomitant systemic disease (hypertension, diabetes, cardiovascular disease, cancer, chronic lung diseases, and other immunosuppressive conditions) (1).

It is stated that chronic diseases accompanying the course of COVID-19 pandemic affect the severity and prognosis of the disease. However, there are only a few publications on the effect of comorbid lung diseases.

In light of these findings, this study was intended to investigate patients with chronic lung diseases and their effects on disease severity, intensive care hospitalization and mortality in patients hospitalized in our hospital.

## Materials and methods

This is a retrospective and observational study covering patients admitted to the Pandemic Clinic between March 11, 2020 and May 31, 2020 (patients with pulmonary diseases, infectious diseases, and those who applied to internal medicine clinics) with a COVID-19 Pneumonia diagnosis at the hospital.

The plan of the study was to record the study parameters of cases from patients’ files in the hospital system, save them as excel data, and conduct statistical analysis. The study was devised to include 1,549 patients. COVID-19 cases aged over 18 were accepted in our study.

Cases with the diagnosis of asthma and chronic obstructive lung disease (COPD) who were followed by internal and pulmonary medicine clinics under the inhaler treatment were recorded. Cases having diagnosis of interstitial lung disease (ILD) before the diagnosis of COVİD-19 Pneumonia were defined as ILD group. Cases who were receiving a treatment for the diagnosis of lung cancer and were being followed. The diagnoses of all the cases were recorded, depending on the files and records in the hospital information system. The cases with asthma, COPD, ILD and lung cancer were grouped as the chronic lung diseases patients.

According to the case definitions suggested by the Turkish Ministry of Health Science Board, patients with possible and definitive cases were included. Patients with positive Real-Time.

Polymerase Chain Reaction (PCR) tests and patients diagnosed with Clinical/radiological COVID-19 were evaluated together (1).

Demographic features of the patients with chronic lung disease (Group 1) and without chronic lung disease (Group 2) were compared in terms of the treatment they **receive**, admission to intensive care, mortality and follow-up parameters such as age, gender, duration of symptoms, additional diseases, smoking status, presence of asthma and Chronic Obstructive Pulmonary Disease (COPD), bronchiectasis, presence of ILD, presence of lung cancer, PCR test results, number of performed PCR tests, severity of the disease in the demographic data (serious or moderate and mild disease), follow-up place (inpatient, intensive care), whether intubation was required or not and the treatments used (Plaquenil, Kaletra, Favipiravir, Tocilizumab, Azithromycin, Oseltamivir, other antibiotic treatments). Hospitalization period, hospitalization time in intensive care unit, routine blood tests (C–reactive Protein, Hemogram, Biochemistry, D-Dimer, Ferritin, Interleukin-6, Fibrinogen, Serum Iron Levels) and hospital information system records were evaluated and analyzed.

**FIGURE 1:**
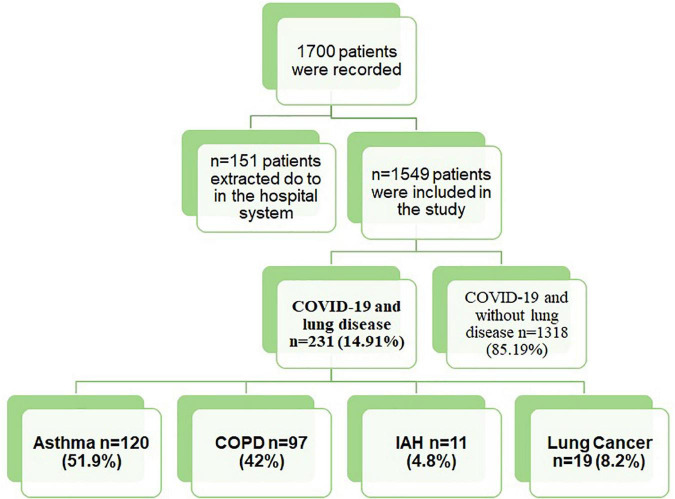
A flow chart of patient recruitment. Abbreviations: COPD, chronic obstructive lung disease; ILD, interstitial lung disease.

The patients excluded from the study had the following criteria: The cases were under the age of eighteen; the ones who did not agree to participate in the study. Also, patients hospitalized in COVID services with missing data in the system were excluded (Figure 1).

### Statistical analysis

All statistical analyses were performed using Statistical Package for the Social Sciences (SPSS) for Windows version 15.0 (SPSS, Chicago, IL, USA). Shapiro–Wilk and Kolmogorov–Smirnov tests were used to assess the assumption of normality. Normally distributed continuous variables were expressed as mean ± standard deviation while the continuous variables that did not have a normal distribution were expressed as median (minimum-maximum). Also, categorical variables were summarized as counts (percentages). Comparisons of continuous variables between two independent groups were performed using Student’s *t*-test and Mann–Whitney *U*-test. Associations between categorical variables were determined by Chi-square test and Fisher’s Exact test. In order to define the risk factors of mortality, risk ratios (RR) were estimated by negative binomial regression model with robust error variances. A two-sided *p*-value < 0.05 was considered to be statistically significant.

## Results

COVID- 19 patients with lung disease (group 1) and those without (group 2) were compared in terms of demographic parameters. Among the comorbidities, diabetes mellitus, coronary artery disease and hypertension were observed with a higher frequency in patients with chronic lung disease than in those without (*p* = 0.001) (Table 1).

**TABLE 1:** Demographic parameter of patients with chronic disease (Group 1) and without chronic lung disease (Group 2).

Variables	COVID-19 and lung disease (*n* = 231) (14.91%)	COVID-19 and without lung disease (*n* = 1318) (85.19%)	*p*
Age (Mean ± SD)	60.85 ± 15.28	49.28 ± 17.59	f<0.001
Male (*n*, %)	122 (52.8)	819 (62.1)	0.007
**Symptoms (*n*, %)**			
Dyspnea	148 (64.1)	366 (27.8)	<0.001
Throat pain	26 (11.3)	197 (14.9)	0.140
Runy nose	2 (0.9)	39 (3)	0.068
Nausea and vomiting	14 (6.1)	81 (6.1)	0.960
Diarrhea	6 (2.6)	79 (6)	0.037
Fever	76 (32.9)	488 (37)	0.229
Cough	129 (55.8)	702 (53.3)	0.468
Sputum	23 (10)	88 (6.7)	0.075
**Comorbidity (*n*, %)**			
Diabetes mellitus	61 (26.4)	187 (14.2)	<0.001
Coronary artery dis.	56 (24.2)	137 (10.4)	<0.001
Hypertension	113 (48.9)	419 (31.8)	<0.001
Kidney dis.	16 (6.9)	56 (4.2)	0.075
Others	22 (9.6)	85 (6.5)	0.371
**Chronic lung disease (*n*,%)**			
Asthma	120 (51.9)	0 (0)	<0.001
COPD	97 (42)	0 (0)	<0.001
Interstitial lung dis.	11 (4.8)	0 (0)	<0.001
Lung cancer	19 (8.2)	0 (0)	<0.001
**Physical examination**			
sO_2_ (Mean ± SD)	91.56 ± 5.51	93.70 ± 3.63	<0.001
Respiratory rate (Mean ± SD)	21.27 ± 4.08	20.37 ± 2.82	0.001
Blood pressure _ Systolic {Mean ± SD [Median (min-max)]}	93.19 ± 24.88 [90 (24–140)]	90.48 ± 21.41 [90 (24–140)]	0.116
Blood pressure _ Diastolic {Mean ± SD [Median (min-max)]}	64.38 ± 10.58 [60 (59–90)]	62.66 ± 8.40 [60 (59–90)]	0.032
qSOFA scores {Mean ± SD [Median (min-max)]}	0.41 ± 0.70 [0 (0–3)]	0.33 ± 0.59 [0 (0–3)]	0.162

Symptom of dyspnea was higher in group 1 than group 2, and oxygen saturation levels were lower in group 1 (*p* = 0.001). Groups were compared in terms of pneumonia severity, intensive care admission and mortality. Group 1 cases were found to receive more oxygen therapy and mechanical ventilation than group 2 cases (Table 2). It was also found that mortality rates were higher than group 2. Groups were compared in terms of laboratory parameters (Table 3).

**TABLE 2:** The severity of pneumonia follow-up parameter of patients with chronic disease (Group 1) and without chronic lung disease (Group 2).

Variables	COVID-19 and lung disease (*n* = 231) (14.91%)	COVID-19 and without lung disease (*n* = 1318) (85.19%)	*P*
**Severity of pneumonia (*n*, %)**			
Severe pneumonia (SARI)	11 (4.8)	83 (6.3)	0.001
Mild disease	65 (28.1)	344 (26.1)	
Critical illness	17 (7.4)	34 (2.6)	
Pneumonia	138 (59.7)	857 (65)	
**Treatment (*n*, %)**			
Oseltamivir	117 (50.6)	456 (34.6)	<0.001
Enoxoparine (Subcutan)	170 (73.6)	704 (53.4)	<0.001
Antibiotic	185 (80.1)	798 (60.5)	<0.001
Intensive care unit follow-up **{Mean ± SD [Median (min-max)]}**	8.92 ± 7.28 [7 (1–27)]	8.92 ± 7.22 [7 (1–30)]	0.843
Intensive care needs developed in the day of hospitalization **{Mean ± SD [Median (min-max)]}**	2.60 ± 2.04 [1.5 (1–7)]	3.78 ± 8.90 [2 (1–90)]	0.651
Mechanic ventilation on the day of hospitalization? **{Mean ± SD [Median (min-max)]}**	3.27 ± 3.88 [1 (1–12)]	3.88 ± 2.75 [3 (1–10)]	0.270
Oxygen (*n*, %)	95 (41.1)	260 (19.7)	<0.001
High flow oxygen (*n*, %)	4 (1.7)	28 (2.1)	1.000
NIMV* (*n*, %)	3 (1.3)	5 (0.4)	0.103
Mechanic ventilation (*n*, %)	11 (4.8)	31 (2.4)	0.038
Mechanic ventilation duration^j^ **{Mean ± SD [Median (min-max)]}**	14.67 ± 11.22 [15 (3–27)]	14.68 ± 7.67 [15 (2–27)]	0.975
ECMO* (n, %)	1 (0.4)	0 (0)	0.149
Mortality (*n*, %)	12 (5.2)	29 (2.2)	0.009

NIMV, non-invasive mechanic ventilation; ECMO, extracorporeal membrane oxygenation.

**TABLE 3:** The laboratory follow-up parameter of patients with chronic disease (Group 1) and without chronic lung disease (Group 2).

Variables	COVID-19 and lung disease (*n* = 231) (14.91%)	COVID-19 and without lung disease (*n* = 1318) (85.19%)	*P*
WBC* x10^9/L (1–3 Day)	7310 (1520–44000)	7000 (1141–59290)	<0.001
Lymphocytex 10^9/L (1–3 Day)	1200 (0–33060)	1435 (0–49620)	0.017
Hgb* g/dL (1–3 ay)	13.40 (6–19)	13.70 (6–19)	0.025
PLT* g/dL (1–3 Day)	231000 (77000–560000)	232000 (61000–607000)	0.514
Urea mg/dL (1–3 Day)	2.5 (0–1394)	2.5 (0–43953)	0.106
Crea mg/dL (1–3 Day)	0.83 (0–7)	0.83 (0–10)	0.550
AST* U/L (1–3 Day)	21 (4–131)	22 (2–1015)	0.041
ALT* U/L (1–3 Day)	20 (6–147)	26 (3–634)	<0.001
CK* U/L (1–3 Day)	79 (2–1229)	90 (1–6197)	0.012
LDH* U/L (1–3 Day)	244 (132–1051)	225 (38–2586)	<0.001
CRP* μ g/L (1–3 Day)	0.04 (0–262)	0.02 (0–506)	0.004
PCT* μ g/L (1–3 Day)	0.05 (0–48)	0.04 (0–142)	0.004
Myoglobin ng/L (1–3 Day)	66 (8–1000)	46 (0–1000)	<0.001
Ferritin μ g/L (1–3 Day)	85.5 (1–15441)	108 (1–9569)	0.005
D-Dimer mg/L (1–3 Day)	0.74 (0–35)	0.50 (0–44)	<0.001
Fibrinogen ng/L (1–3 Day)	4.01 (2–43)	3.53 (0–92)	0.001
IL-6* pg/mL (1–3 Day)	11.60 (2–4399)	8.96 (1–4405)	0.180

*Cells represent median (min-max).

WBC, white blood cell; Hgb, hemoglobin; PLT, platelet; AST, aspartate aminotransferase; ALT, alanine aminotransferase; CK, creatine kinase; LDH, lactic dehydrogenase; CRP, C-reactive protein; PCT, procalcitonin; IL-6, Interleukin-6.

Subgroup analysis was performed in terms of chronic lung diseases. There was no significant difference between patients with and without asthma in terms of severity of pneumonia, admission to intensive care unit and mortality. Patients with and without COPD were compared end critical illness, pharmacotherapy, oxygen therapy, and mortality were found to be significantly higher in patients with COPD than those without (*p* ≤ 0.001). There was no significant difference between the groups with and without ILD. There was only a significant difference in mortality between patients with and without ILD [*n* %, 2 (18.2), 39 (2.5), *p* = 0.03)]. Patients with and without lung cancer were compared and there was only a significant difference in mortality [*n* %, 3 (15.8), 38 (2.5), *p* = 0.01)].

**FIGURE 2:**
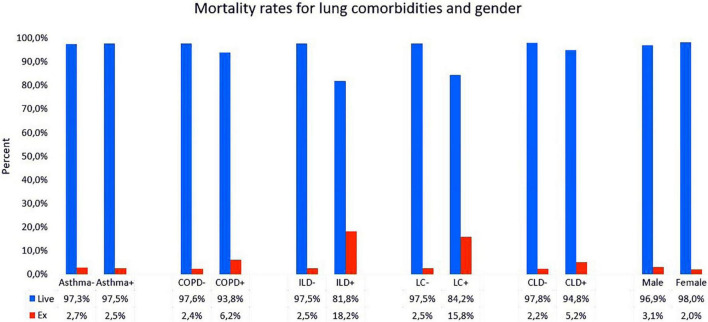
Mortality rates for lung comorbidities and gender in patients with chronic disease (Group 1) and without chronic lung disease (Group 2). Abbreviations: COPD, chronic obstructive lung disease; ILD, interstitial lung disease; LC, lung cancer; CLD, chronic lung disease.

Univariate and multiple logistic regression analyses were performed in order to define the risk factors of mortality. In univariate analysis, COPD, ILD, and lung cancer were observed as associated with higher mortality as COPD, ILD and lung cancer OR were [OR 2.66 CI95% (Min–Max, 1.094–6.511) *p* = 0.02], [OR 8.54 CI 95% (Min–Max, 1.786–40.842) *p* = 0.03], [OR 7.36 CI95% (Min–Max, 2.058–26.332) *p* = 0.001] respectively. Also, advanced age, male gender and presence of chronic lung disease were found to be significantly associated with mortality in multiple logistic regression analysis (*p* = 0.001). It was determined that patients with chronic lung disease had 25.76-fold increased risk of mortality [OR 25.76 CI 95% (Min–Max, 2.445–271.465) *p* = 0.007] (Figure 2).

## Discussion

Upon the evaluation of the patients who applied to our pandemic clinic, it was observed in our study that patients with concomitant chronic lung disease had a poor prognosis than those without. It was shown that early hospitalization was higher in chronic lung disease patients those without lung disease.

While the rate of hospitalization during COVID pandemic was 4.6 per 100.000, this rate was 13.8% in cases over 65 years of age. This rate was 12% (*n* = 178) in adults as of March 30. Comorbidities were found in 89.3% of these cases. The most common comorbidities were HT with 49.7%, obesity with 48.3%, DM with 28.3% and chronic lung diseases with 34.6%. In light of these findings, multiple comorbidities were among the most important factors in hospitalization in adults (5, 6). In our study, the most common comorbidities accompanying patients with chronic lung disease were hypertension *n* = 113 (48.9%), while the second and third ones were diabetes mellitus *n* = 61 (26.4%) and coronary artery disease *n* = 56 (24.2%).

In addition to chronic systemic diseases, accompanying cases of asthma, COPD and emphysema were seen most frequently among chronic lung diseases (7, 8).

In the series in which 74.439 cases were evaluated, the presence of chronic lung diseases was observed in *n* = 656 (9.2%) cases (2). In these series, asthma and COPD cases were evaluated in this group. It was shown that 15% of these cases were followed up in the service and 21% in intensive care (2). According to Centers for Disease Control and Prevention (CDC) data of 2018 in the United States, asthma was 7.9% (9) in adults and COPD was 5.9% (10). COVID-19–associated hospitalization rates for patients admitted during March 2020 in US were as follows: Among 1.482 patients hospitalized with COVID-19, patients with Asthma were 17% and patients with COPD were 10.7% (10). Of the 1549 cases in our study, 231 cases (14.91%) had chronic lung disease. Of these, 120 (51.9%) patients had asthma, 97 (42%) had COPD, 11 (4.8%) had ILD, and 19 (8.2%) patients had lung cancer.

Recent meta-analyses have demonstrated an almost six times increase in the odds of mortality for patients with COPD and a 2.5 times one for those with diabetes, possibly due to the underlying pulmonary and immune dysfunction (7, 11–13). In our study, 2.66, 8.54, and 7.36 higher mortality risks were found in patients with COPD, ILD and lung cancer, respectively. However, there was no increase in mortality risk in asthmatic patients.

Also, in another analysis evaluating COVID-19 and comorbidities, chronic respiratory diseases were found to be 1.8%. When severe and mild cases of pneumonia were compared, it was shown that accompanying diseases were seen 2.46 times more in severe cases (OR 2.46; 95% CI; 1.76–3.44). In general, it has been shown that patients with severe course were older and had more comorbidities (7). In a study with influenza, the relationship between the severity of disease and comorbidities was investigated. Accordingly, it was found that cases with severe Pneumonia with COPD were at 1.49 times (OR 1.49, 95% CI: 1.10–2.01) higher mortality risk than mild cases (7, 13). In our study, cases with COPD were significantly higher in patients with critical disease than those without (*p* = 0.001).

The presence of respiratory disease has been shown to have a similar effect in patients with MERS (14). In our study, in cases with COVID-19 pneumonia, the presence of chronic lung disease, advanced age and male gender were found to be significantly associated with mortality.

In a review published on this subject, COVID-19, which is considered to have fibrotic ILD, involves the risk of transmission in the diagnostic process, and the necessary tests such as respiratory function test and bronchoscopic biopsy should be evaluated. It has been reported that they should be performed if there is an absolute indication in terms of diagnosis (2, 3). In addition, if a treatment decision that will affect the course of the disease is required in a patient monitored for COVID-19, it is recommended to perform procedures such as invasive bronchoscopic biopsy.

It was hypothesized that severe COVID-19 infection could lead to an exaggerated immune response. It remains unknown if the physician used initiation or maintenance of immunomodulatory therapies for patients with fibrotic ILD during COVID-19 Pneumonia. Also, there is no evidence that anti-fibrotic therapies impact the risk or severity of COVID-19 infection (15–21). In our study, a significant difference was noted in terms of mortality between patients with and without ILD.

Pulmonary Embolism is reported in COVID-19 cases. Moreover, in postmortem biopsies, the presence of microthrombus in small pulmonary vessels in the lung and occlusion of the pulmonary vessels have been shown (22, 23). In our study, pulmonary embolism was detected in 6 cases and one was given thrombolytic therapy.

In the COVID-19 pandemic, cancer patients are regarded as a highly vulnerable group. Active cancer cases have more serious risks because they are immunosuppressed due to the chemotherapy and radiotherapy they receive as treatment. Therefore, if they have COVID-19 pneumonia, the treatment protocol they receive should be postponed (24), The clinical characteristics of COVID-19 infected cancer patients remain largely unknown. Anemia and hypoproteinemia were considered to be major consequences of nutritional deterioration in cancer patients according to several studies (25). In the general COVID-19 infected population, 4.7% of confirmed patients reached a clinically critical status, and 2.3% of critical cases ending in fatality (3). Early case series from US, China and Italy have suggested that patients with malignancy are more susceptible to severe infection and mortality from COVID-19 (26–29), In a national analysis of China with 1590 cases, it was found that the presence of malignancy exacerbates the course of COVID-19 3.5 times (5).

Among the malignancies within the US population there was 55% mortality among lung, 14% among breast, 20% among prostate, and 38% among colorectal cancer patients in a large-scale case study (30). The data in this study showed that comorbid diseases accompanying cancer and COVID diagnoses were significantly higher in patients who died due to chronic lung diseases, congestive heart failure and coronary artery diseases than those living. It was observed that the mortality rate increased in cases with malignancy compared to those without.

Prone position and high-flow oxygen therapy were applied in cases whose hypoxia continued despite high-dose nasal oxygen therapy while following up the cases with COVID-pneumonia. In the literature, the benefit of high-flow oxygen therapy in COVID-19 pneumonia is discussed in studies comparing both treatments (31).

In our study, it was reported that the comorbidity of asthma and bronchiectasis in cases with COVID did not adversely affect the prognosis. In the literature, it has been reported in previous publications that asthma and bronchiectasis are worse prognostic factors (32–34). However, in our study, it was not possible to obtain the severity of the asthmatic cases from the file information and whether bronchiectasis was accompanied in the same case. In addition, it is not clear whether the main factor contributing to the clinical severity of the patient is related to the pathogenesis of COVID-19 or to the severity of concomitant bronchiectasis or asthma.

Our study has some restrictive points as cases of asthma, COPD, and bronchiectasis diagnoses were based on file and hospital follow-up. Evidence-based tests such as respiratory function tests and allergic skin tests required for diagnosis were currently not available due to pandemic conditions. Diagnoses based on clinical, laboratory and radiological data and records in the hospital data system were analyzed. According to the pandemic guidelines, tests such as respiratory function tests and allergic skin tests could not be updated. Although mortality was higher with COPD, ILD and lung cancer in our study, there is a need to confirm these results with larger cohort studies on this subject.

## Conclusion

The presence of chronic lung disease, advanced age and male gender was found to be significantly associated with mortality in our study. In chronic lung diseases, the mortality rate of cases with COPD, lung cancer and ILD was found to be significantly higher than those without. This is the first study investigating the relationship between chronic lung diseases and pneumonia severity and mortality. However prospective cohort studies evaluating the relationship between chronic lung disease and COVİD-19 Pneumonia should be conducted in the future.

## Data availability statement

The original contributions presented in this study are included in the article/supplementary material, further inquiries can be directed to the corresponding author.

## Ethics statement

Ethical review and approval was not required for the study on human participants in accordance with the local legislation and institutional requirements. Written informed consent from the patients/participants or patients/participants legal guardian/next of kin was not required to participate in this study in accordance with the national legislation and the institutional requirements.

## Author contributions

HK, EA, AgK, AS, and EU: conceptualization. HK, EA, AgK, and EU: methodology. DG and SH: software, validation, and formal analysis. HK, EA, AgK, EU, RG, BK, İA, MC, EhA, EP, FS, YK, SG, EM, ME, AD, HE, ZH, AK, FE, AA, EgA, SK, EG, EK, Oİ, AY, BG, EC, İT, DG, SH, and AS: investigation and resources. HK, EA, AgK, EU, RG, BK, İA, MC, EhA, EP, FS, YK, SG, EM, ME, AD, HE, and ZH: data curation. HK, EA, and AgK: writing. HK, EA, AgK, and AS: visualization. AgK and AS: supervision. HK: project administration. All authors contributed to the article and approved the submitted version.
